# Printing Highly Controlled Suspended Carbon Nanotube Network on Micro-patterned Superhydrophobic Flexible Surface

**DOI:** 10.1038/srep15908

**Published:** 2015-10-29

**Authors:** Bo Li, Xin Wang, Hyun Young Jung, Young Lae Kim, Jeremy T. Robinson, Maxim Zalalutdinov, Sanghyun Hong, Ji Hao, Pulickel M. Ajayan, Kai-Tak Wan, Yung Joon Jung

**Affiliations:** 1 Department of Mechanical and Industrial Engineering, Northeastern University, Boston, MA 02115, USA; 2 Department of Materials Science and NanoEngineering, Rice University, Houston, TX 77005, USA; 3 George J. Kostas Research Institute for Homeland Security, Northeastern University, Boston, MA 02115, USA; 4 Department of Energy Engineering, Gyeongnam National University of Science and Technology, Jinju, Gyeongnam, 660-758, South Korea; 5 Naval Research Laboratory, Washington, D.C. 20375, USA

## Abstract

Suspended single-walled carbon nanotubes (SWCNTs) offer unique functionalities for electronic and electromechanical systems. Due to their outstanding flexible nature, suspended SWCNT architectures have great potential for integration into flexible electronic systems. However, current techniques for integrating SWCNT architectures with flexible substrates are largely absent, especially in a manner that is both scalable and well controlled. Here, we present a new nanostructured transfer paradigm to print scalable and well-defined suspended nano/microscale SWCNT networks on 3D patterned flexible substrates with micro- to nanoscale precision. The underlying printing/transfer mechanism, as well as the mechanical, electromechanical, and mechanical resonance properties of the suspended SWCNTs are characterized, including identifying metrics relevant for reliable and sensitive device structures. Our approach represents a fast, scalable and general method for building suspended nano/micro SWCNT architectures suitable for flexible sensing and actuation systems.

Carbon nanotubes (CNTs) are argued to be an ideal candidate for future electronic and electromechanical systems^1^,^2^,^3^,^4^,^5^,^6^. Single-walled carbon nanotubes (SWCNTs) consist of a seamless layer of sp^2^-bonded carbon atoms and as such, are ‘all-surface’ and are influenced by any supporting substrates^7^,^8^,^9^,^10^,^11^,^12^,^13^. For example, a CNT thermal fluidic sensor is based on a pronounced temperature dependent resistivity. Once embedded into a substrate, the thermal energy generated on a CNT will partly dissipate into the substrate, leading to a compromised precision and sensitivity of the sensor^14^. When suspended, the influence of the substrate is eliminated and the intrinsic nanotube properties are accessible. For example, suspended SWCNT devices show high mobility and high electrical and thermal conductivities^8^,^10^,^11^. More importantly, suspended nanotube architectures can enable a wide range of new functionalities such as hysteresis-free transistors^15^, high-frequency resonators^16^,^17^,^18^, sensors or detectors^19^,^20^,^21^, actuators (*e.g.* artificial muscle)^22^, atomic level balances^23^, switches for memory systems^24^, bolometers^25^, illuminators^22^, and superconducting quantum interference devices^26^.

Many existing technologies, such as high-temperature growth^27^,^28^, electrophoretic assembly^29^, backside etching^16^, contact printing^30^,^31^ and transfer-etching^32^, are limited to rigid substrates and are not feasible for building large-scale and tightly-controlled suspended CNT structures on flexible substrates. For example, it is not feasible to expose polymeric substrates to the typical high temperatures (>600 °C) required for CNT growth. Therefore, one effort is to reduce the growth temperature to the safety range of polymer. Though CNT growth on polymer substrates has not yet been presented, photo-thermal chemical vapor deposition reducing the temperature of CNT growth to 370 °C, shows significant potential in this direction^33^,^34^. Alternatively, the electrophoretic and backside etching methods that evolved from conventional micro-electro-mechanical system (MEMS) and nano-electro-mechanical system (NEMS) technology are more compatible with current industrial processes and have relatively good controllability in size and position^16^,^29^. However, this high controllability is achieved at the cost of complicated and long fabrication processes. Additionally, the high controllability cannot be readily applied to polymer substrates since precise etching techniques for polymer are limited.

In this paper we demonstrate a new, scalable and versatile strategy for fabricating suspended nano/micro SWCNT networks on micro-patterned flexible polymer substrates. This transfer process is made possible through understanding the interactions between micro-patterned polymer surfaces, solutions, and assembled SWCNTs and by controlling their solid-liquid-vapor (SLV) interfacial behavior. Our method is inspired by the superhydrophobic Lotus leaf surface, where water droplets are suspended on top of microscale papillae that leaves air pockets trapped beneath the suspended droplets, forming a SLV interface with reduced or zero capillary forces^35^,^36^,^37^,^38^,^39^. By combining a micro-patterned, Lotus leaf-like substrate with a wet-contact printing transfer technique, almost 100% of SWCNT networks could be suspended without collapsing from capillary forces. Thus, this transfer approach is highly effective in “printing” nano/microscale SWCNT suspended networks on patterned superhydrophobic polymer surfaces that can then be used for 3D flexible electronic devices including NEMS and MEMS applications. Moreover, this entire transfer technique could be conducted under a one-step, CMOS compatible process, suitable for Roll-to-Roll manufacturing.

## Results

**Figure 1:**
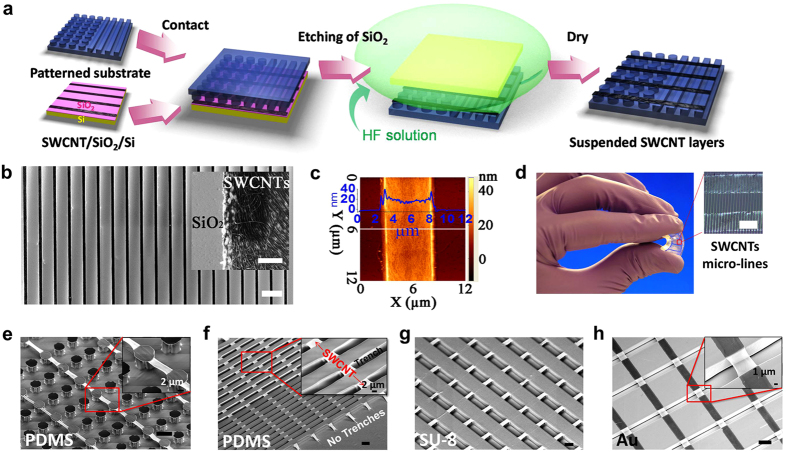
Approach for wet-contact printing and suspended SWCNT micro-lines on micro-patterned polymer substrates. (**a**) Schematics of the wet-contact printing process. (**b**) Large-scale assembly of arrays of SWCNT micro-lines (black lines, 6 μm in width) on SiO_2_/Si substrate and the edge of one SWCNT micro-line showing sharp boundary between SiO_2_ surface and SWCNTs network (inset). (**c**) AFM image of an as-assembled SWCNT micro-line with center thickness of ~15 nm. Inset, a line profile along the white line in (**c**). (**d**) A flexible suspended SWCNT-PDMS sample under bending condition and optical image of two SWCNT micro-lines (100 μm in width) after bending. PDMS micro-lines are 7.5 μm in width and 15 μm in center distance. (**e**) PDMS substrate with micro-pillars (6 μm in diameter, 18 μm in center to center distance), (**f**) PDMS substrate patterned with arrays of micro-lines (6 μm in width, 9 to 12 μm in center distance), (**g**) SU-8 substrate patterned with arrays of micro-lines (20 μm in width, 30 μm in center distance), and (**h**) 150 nm-thick Au coated SU-8 substrate patterned with arrays of micro-lines (30 μm in width and 36 μm in center distance). A very thin layer (several-nanometer thick) of Au/Pd coating is required for imaging SWCNT on PDMS (**e,f**) and SU-8 (**g**) to eliminate the charging of polymer substrates. The heights of all the micro-patterns are 6 μm. The scalar bars are (**b**) 40 μm (500 nm for inset), (**d**) inset 100 μm (**e**)10 μm (2 μm for inset), (**f**) 10 μm (2 μm for inset), (**g**) 10 μm, and (**h**) 10 μm (1 μm for inset).

Inspired by the superhydrophobic Lotus leaf surface^35^,^36^,^37^,^38^,^39^, our transfer process (termed “wet-contact printing method”) is schematically shown in Fig. 1a. First, SWCNT network films are patterned (*e.g.*, lines, squares, and films) on a SiO_2_ (100 nm)/Si substrate using a template guided fluidic assembly process^40^,^41^,^42^,^43^,^44^, where the SiO_2_ layer acts as a sacrificial release layer. As shown in Fig. 1b,c, we pattern arrays of SWCNT micro-lines on SiO_2_/Si substrate, where the length of the features can be defined precisely through the lithography process. The thickness of SWCNT micro-lines is around 15 nm in the center (Fig. 1c). To form suspended SWCNT structures, we use a Lotus leaf-like patterned polymeric receiving substrate with arrays of micro-lines or pillars (*e.g*., polydimenthylsiloxane (PDMS), epoxy resin (SU-8), or poly(methyl methacrylate) (PMMA)), and bring it into dry contact with the SWCNT networks on SiO_2_/Si substrates. The joined substrates (e.g., PDMS/SWCNT/SiO_2_/Si) are then submerged in a dilute hydrofluoric (HF) acid solution (16 wt.%), which etches the SiO_2_ layer and releases the SWCNT architectures onto the patterned polymeric substrates. Finally, the residual HF acid solution is removed by simply tilting the superhydrophobic substrate, leaving the SWCNT networks suspended between nano/micro patterns of polymers. The Raman spectrum of SWCNT micro-line (D/G intensity ratio is ~0.1, see Supplementary Fig. S1) on donor substrate confirms good quality of SWCNTs. The transmission electron microscopy (TEM) image and the energy-dispersive X-ray spectroscopy (EDX) of the SWCNTs transferred to TEM grid demonstrate that HF etching does not significantly damage or dope the SWCNTs (see Supplementary Fig. S2).

These suspended SWCNT architectures are mechanically flexible and optically transparent. Figure 1d shows a bending PDMS substrate (micro-lines patterned, 7.5 μm in width, 6 μm in height and 15 μm in center distance) with suspended SWCNTs micro-lines (100 μm in width). After extensive bending tests (strain up to ε = 26%, see Supplementary Fig. S3), the suspended SWCNT micro-lines remain intact after releasing (Fig. 1d, inset), where neither large cracks nor breaking films are observed. Our SWCNT films show better flexibility when compared to thin indium tin oxide (ITO) films (80 nm thick), which start to crack at ε = 1.59%^45^. Figure 1e–g show representative tilted angle SEM images of transferred SWCNT networks suspended on various micro patterned soft (PDMS) and hard (Epoxy, SU-8) polymer substrates. Figure 1e highlights the unique 3D micro-architecture, where SWCNT micro-lines (width = 4 μm) are precisely aligned over arrays of PDMS pillars (diameter = 6 μm; height = 6 μm). In Fig. 1f, arrays of SWCNT micro-lines (width = 6 μm) are suspended over PDMS line patterns (width *x*  = 6 μm and center distance *y* = 9 and 12 μm from right to left). As shown in Fig. 1g, we are able to construct suspended SWCNT networks on patterns of high surface energy polymers such as the epoxy resin based photoresist (SU-8). Furthermore, the process works for Au-patterned surfaces, a necessary step in fabricating semi-transparent SWCNT networks for flexible electronic device structures (Fig. 1h). The successes of suspending SWCNT architectures on different substrates and micro-patterns suggest the great scalability of this wet-contact printing method.

**Figure 2:**
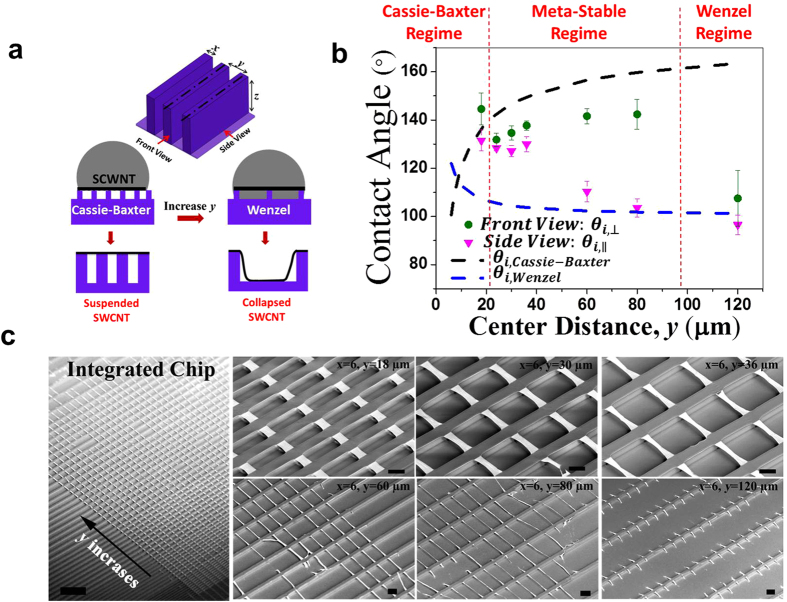
The influence of substrate geometry on suspended SWCNT architectures. (**a**) The schematic illustrating superhydrophobic (Cassie-Baxter mode) and complete wetting (Wenzel mode) water-polymer interfaces on micro-lines patterned polymeric substrate. The structure parameter of micro-lines: the width (*x*) and the distance between the center of neighbor micro-lines (*y*), the height of micro-lines (*z*). (**b**) Dependence of apparent contact angle (*θ*_*i*_) on center distance (*y*) ranging from *y *= 18 to 120 μm for micro-patterned PDMS substrate with the constant line width (*x *= 6 μm). The theoretical contact angles calculated from Cassie-Baxter equation (*θ*_*i*_,_*Cassie−Baxter*_) and Wenzel equation (*θ*_*i*_,_*Wenzel*_) are plotted as black dashed line and blue dashed line, respectively. The front view apparent contact angles are plotted as olive dots (*θ*_*i*⊥_) and the side view apparent contact angles (*θ*_*i*_,_‖_) are plotted as pink triangles. (**c**) The resultant SWCNT architectures obtained through wet-contact printing method over the substrates shown in (**b**). Perfectly suspended SWCNT micro-lines (6 μm in width) can be obtained until the substrate with *y *= 36 μm. SWCNT architectures begin to miss and collapse for the sample with *y *= 60 μm and 80 μm. Still, large amount of suspended architectures remain. For *y *= 120 μm, no suspended SWCNT architectures can be obtained. The scalar bars in (**c**) are 100 μm for integrated chip on the left and 10 μm for the rest of SEM images.

The formation of suspended SWCNT architectures is a result of the forces balanced at the SLV interface between the SWCNTs, the solution and the substrate. An *in-situ* nanoparticle probing experiment, performed over the micro-line patterned substrate (Fig. 1f), shows that the solution does not wet the micro-trenches (see Supplementary Fig. S4). As such, during our transfer process no solution diffuses underneath the SWCNTs, resulting in zero capillary forces that can collapse the suspended SWCNT architectures to the substrate. To further understand the SLV interface, we designed polymer substrates with different pattern densities, where the width (*x* = 6 μm) and height (*z* = 6 μm) are kept constant, and the pattern spacing, *y*, varies from 18 to 120 μm (Fig. 2a). The measured contact angle for aqueous solutions on these patterns is shown in Fig. 2b and the resulting transfer of SWCNT micro-networks is shown in Fig. 2c and Supplementary Fig. S5. For patterned substrates, there are two distinct wetting modes as shown in Fig. 2a: Cassie-Baxter mode and Wenzel mode. In Cassie-Baxter mode, the solution is suspended over the nanopatterns leaving an air-pocket between solution and the bottom surface of the substrate^46^. A notable example is the Lotus leaf surface, where the solution is strongly expelled by the hydrophobic surface^35^,^36^,^37^,^38^,^39^. Alternatively, in Wenzel mode, the solution completely wets the substrate surface^47^. A transition from Cassie-Baxter mode to Wenzel mode would be expected with the increasing spacing of *y*, and correspondingly a transition from fully suspended to fully collapsed SWCNT architectures will occur.

Experimentally measured contact angles are presented in two different forms, front view (*θ*_*i*⊥_, olive dots) and side view (*θ*_*i*_,_‖_, pink triangles), due to the anisotropic wetting of solution over the anisotropically patterned PDMS substrate (arrays of micro-lines)^48^,^49^,^50^. It should be noted that *θ*_*i*⊥_ is larger than *θ*_*i*_,_‖_ due to the pinning effect at the edges of polymeric patterns^48^. We observe a clear transition from superhydrophobic suspension (Cassie-Baxter mode, *y* = 18 μm) to complete wetting (Wenzel mode, *y* = 120 μm) of solutions on the hydrophobic patterns. Both *θ*_*i*⊥_ and *θ*_*i*_,_‖_ are close to the theoretical predictions of *θ*_*i,Cassie−Baxter*_ when *y* = 18 μm and both values are close to *θ*_*i,Wenzel*_ when *y* = 120 μm, as shown in Fig. 2b (also see Supplementary Fig. S2). Correspondingly, fully suspended and collapsed SWCNTs arrays can be found in SEM images (Fig. 2c). This comparison matches very well with our prediction in Fig. 2a, indicating that the key role of suspending SWCNT architectures on patterned superhydrophobic surface is the elimination of capillary force between suspended SWCNT and patterned substrate.

**Figure 3:**
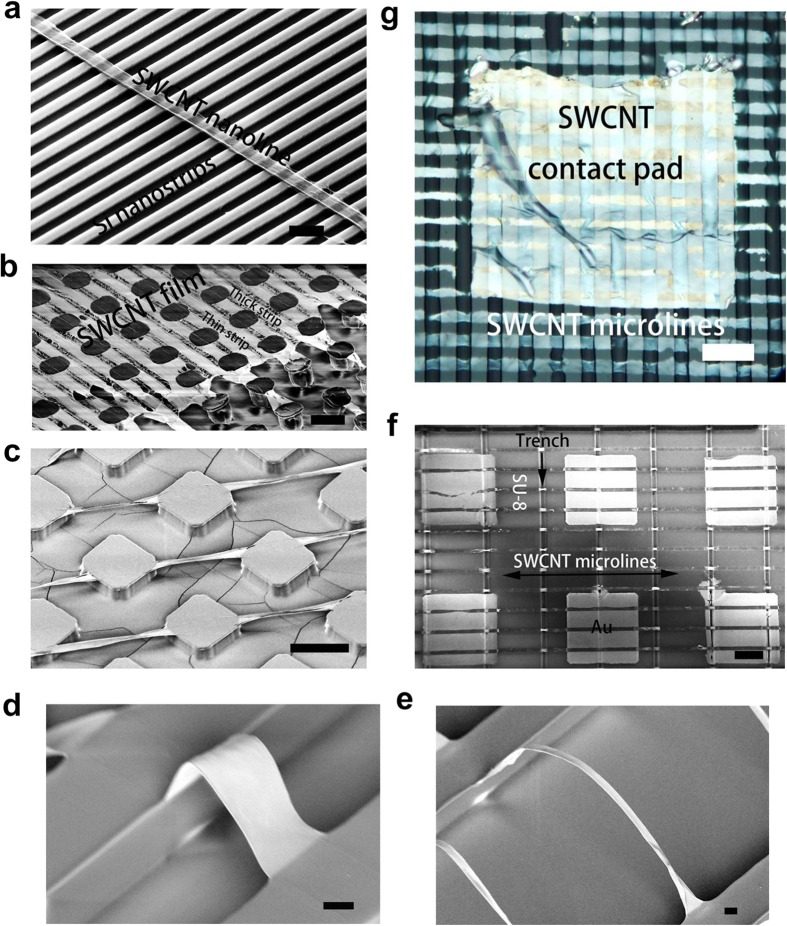
Suspended nano- to macro-scale SWCNT architectures and other capabilities. (**a**) SEM image of 500 nm-wide SWCNT network suspended on Si nano-lines (*x *= 300 nm, *y *= 600 nm and *z *= 200 nm). (**b**) SEM image of macroscale SWCNT film with alternating thick (white) and thin (white and gray) layer suspended on micro-pillars patterned PDMS substrate. (**c**) SEM image of suspended SWCNT suspended with the controlled angle over square-shaped micro-pillars. SEM images of SWCNT micro-arches formed after releasing the pre-stretched PDMS substrate: over 12 μm trench (**d**) and 54 μm trench (**e**), respectively. (**f**) Device level integration of suspended SWCNT micro-lines (6 μm in width) on SU-8 substrate (with 6 μm wide trench) with Au contact pad (100 × 100 μm^2^). Au contact pads were deposited on SU-8 substrate before transferring SWCNT micro-lines. (**g**) Optical microscopy image of all-SWCNT devices obtained by multiple transferring of different SWCNT structures: arrays of micro-lines (6 μm in width) and square pads (100 × 100 μm^2^). Scalar bars: (**a**) 1 μm; (**b**) 10 μm; (**c)** 20 μm; (**d,e**) 2 μm; (**f**) 40 μm and (**g**) 20 μm.

By appropriately designing the pattern density and geometry of receiving polymeric substrates, we could also create a variety of suspended 3D nanotube architectures that were not previously possible. Figure 3 demonstrates the significant potentials of this transfer strategy in terms of scalability (nano- to macro-), for use with other inorganic materials, and in the control of the alignment, morphology, and strain of the suspended SWCNT networks. For example, a SWCNT network (500 nm in width, hundreds of micron in length) could be suspended on silicon line patterns (300 nm in width, 200 nm in height and 600 nm in spacing) using our wet-contact printing transfer process (Fig. 3a). The transfer method also works for complex, micro-textured structures (Fig. 3b), as well as multiple transfers at various deposition angles (Fig. 3c). In addition, using the flexible nature of supporting polymeric substrate, elastic strains of the suspended SWCNT network can be controlled. Figure 3d,e show arrays of arched suspended SWCNT network formed due to the induced-compressive stress by stretching (33% pre-strain) the patterned polymer substrates prior to transfer. After the transfer process, the substrate was released to its original dimension resulting in uniformly compressed arrays of suspended SWCNT networks. This compressive-strain-induced arched nanotube structure may find an immediate application in flexible electronics since at least 33% strain can be achieved without breaking the SWCNT network. As shown in Fig. 3f, a device-level integration of suspended SWCNT networks was achieved on a heterogeneously patterned substrate with gold contact pads and SU-8 micro-trenches (6 μm in width and depth). Finally, we successfully fabricated all-SWCNT 3D devices (Fig. 3g) using a two-step transfer process. For this, SWCNT micro-lines (6 μm in width, centimeter in length) were suspended on PDMS micro-line patterns (*x* = 6, *y* = 12 and *z* = 6 μm). Then, a 100 × 100 μm^2^ SWCNT contact pad was transferred on the top surface of this 3D structured substrate using the same transfer procedures.

**Figure 4:**
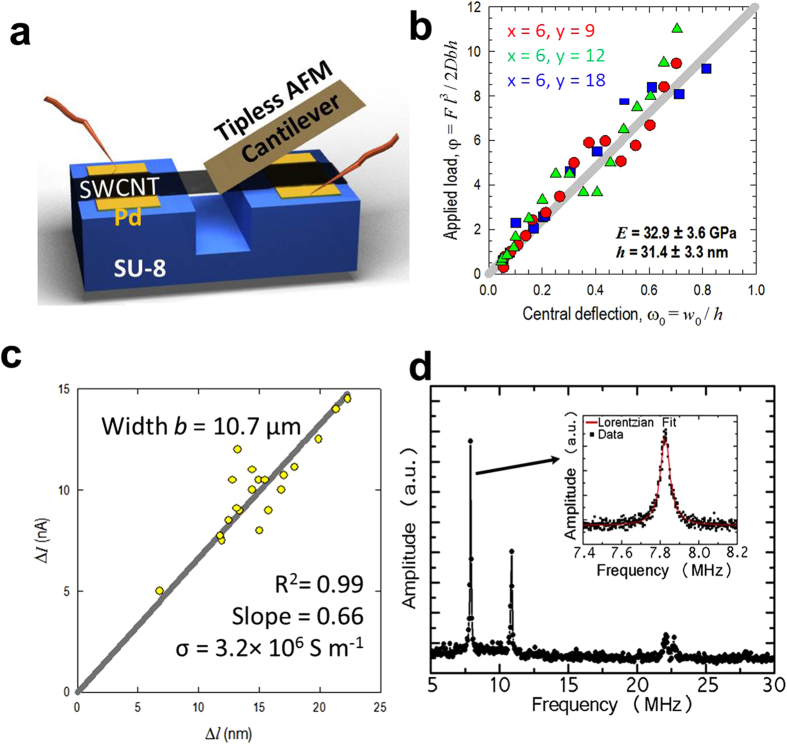
The physical properties of suspended SWCNT micro-lines and its resonating properties. (**a**) Schematic of a tipless AFM cantilever fabricated by focused ion beam. For the electromechanical test, 50 nm-thick Pd was deposited on SU-8 substrate as the contact pads for SWCNT network and electrical bias was applied to the SWCNT through contact pads during the indentation process. (**b**) The dependence of applied load versus central deflection of SWCNT film. (**c**) The measured change of current (Δ*I*) with respect to calculated indentation depth (Δ*l*) of suspended SWCNT micro-lines (widths *b *= 10.7 μm). (**d**) Resonance response of a suspended SWCNT micro-line (6 μm in width) bridge over a 10 μm trench.

In order to understand mechanical and electromechanical properties of these suspended SWCNT networks, we applied a line load using an atomic force microscope (AFM) while simultaneously measuring electrical resistance of the suspended networks (Fig. 4a). Great repeatability and no hysteresis are found during sequential mechanical indentations and contractions (see Supplementary Fig. S6). From the calculated force-displacement curves (Fig. 4b), a clear transition from bending to stretching domain is observed. The calculated Young’s modulus of the suspended SWCNT networks is 32.9 ± 3.6 GPa. This is among the highest values reported for carbon nanotube networks (*E* = 17–35 GPa)^51^,^52^ and exceeds the value for graphene paper (*E* = 31.70 GPa)^53^. A linear decrease in current (Δ*I*) with respect to the network elongation (Δ*l*) is observed for all suspended SWCNT films tested. The experimental details and calculations can be found in Supplementary Fig. S7. The linear dependence demonstrates constant electrical conductivities (around 3 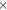 10^6^ S/m) during elastic deformation of networks and suggests that the suspended SWCNT architecture could be used as microsensor for motion or deformation.

To further validate the potential of these suspended SWCNT networks as resonating units in MEMS/NEMS applications, we also measured their vibrational properties using a well-established technique of optical interferometry^54^,^55^. Figure 4d shows a representative resonance response of a SWCNT network bridge over a 10 μm span. Several resonance peaks appear, with the fundamental frequency *f*_*o*_ = 7.83 MHz and quality factor *Q* = 205 (inset). The quality factor is a measure of the energy loss or dissipation within the system defined by *Q *= *f*/Δf, where Δ*f* is the full width half maximum (FWHM) of the resonance response. The average quality factor of 16 different resonators is approximately 210 with a standard deviation of 62. In a continuum mechanics approximation, the flexural resonance frequencies for a doubly clamped beam are given by 
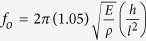
, where *E* is the Young’s modulus, ρ is the density, *h* is the thickness and *l* is the suspended length^56^. Based on the AFM mechanical measurements, *f*_*o*_ was calculated as 8.14 MHz, which is close to our measured results, given *E *= 32.9 GPa, ρ* *= 2.13 g/cm^3^ (SWCNTs are densely packed and a high reference value is chosen^57^), *h *= 31.4 nm and *l *= 10 μm.

## Discussion

To incorporate our process into advanced manufacturing schemes, it is essential to understand the mechanisms underlying the transferring technique. During the transferring process, the SWCNTs, the patterned substrate and the solution are in direct contact, and the forces exchanged at these interfaces are key to understanding the fundamental transfer mechanism. As we know, the wetting process of solution at such interface is strongly influenced by the geometry of the substrate. The wetting behavior of Cassie-Baxter mode can be predicted by theoretical calculations from Cassie-Baxter equation^46^. The relation between apparent contact angle, (*θ*_*i*_) (measured on nano-patterned substrates) and true contact angle, 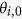, (measured on the flat substrate) is given by Cassie-Baxter equation as follows,


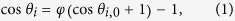


where *φ* is the area fraction of substrate that is in contact with the solution over the projected area (*A*_*0*_)^3^.The relation between the apparent contact angle, *θ*_*i*_, and true contact angle, *θ*_*i,0*_, in the Wenzel equation based on the modified Young’s equation can be described as^47^,


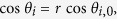


where *r* is the ratio of actual surface area (*A*) and the projected area (*A*_*0*_)^4^. *φ* = *x/y* and *r* *=* (*y* + 2*z*) for line patterns as defined in Fig. 2a. The theoretical prediction of apparent contact angles for superhydrophobic condition (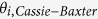) and complete wetting condition (*θ*_*I,Wenzel*_) on PDMS line patterns are plotted as black and blue dashed lines respectively (Fig. 3b).

A transition regime, between where *θ*_*i*_,_‖_ branches from *θ*_*i*⊥_ (from *y *= 24 to *y* = 80 μm), can be defined. In the transition regime, a range of SWCNT network structures are observed with increasing *y*, from well-defined suspended to missing and collapsed structures (Fig. 2c). These results highlight that, in general, the polymer should be designed with narrower trenches and more densely packed patterns in order to obtain a higher yield of suspended architectures. In addition, the active role of SWCNT should be considered here. Since the SWCNT network is strong and robust (*e.g*., Fig. 4), it has some resistance to any residual capillary forces that may arise in cases where some solution is trapped underneath the suspended SWCNT^58^. Taken together, we are able to build very large-scale SWCNT architectures suspended on flexible 3D micro-patterned substrates (center spacing, y,) up to 80 μm.

In conclusion, we have demonstrated a facile, yet powerful and rational approach for the design and fabrication of a variety of complex 3D architectures of SWCNTs-patterned polymer substrates by tailoring interactions between a micro-patterned polymer surface, etching solution, and SWCNTs and by controlling the solid-liquid-vapor interfacial behaviors during the transfer process. To our knowledge, this is the first report resulting in the scalable fabrication of suspended SWCNT nano/micro networks on patterned flexible substrates with almost 100% yield. The transfer strategy offers benefits such as the control over alignment, morphology, and strain in suspended SWCNT networks and suitability for integration with various flexible electronic devices, including flexible sensors, NEMS, and MEMS applications.

## Methods

The SWCNT-de-ionized (DI) water dispersion (0.23 wt%) was purchased from *Brewer Science* Inc. (CNTRENE^TM^ C100). The nanotubes were CVD grown with mixed chirality in nature. The average diameter of SWCNT is about 1 nm with typical length between 0.8 μm and 1 μm. The detailed characterization of SWCNT can be found in REF^44^. Polydimenthylsiloxane (PDMS, Sylgard@184) was purchased from Dow Corning corp. Epoxy resin (SU-8, 2007), poly(methyl methacrylate) (Nano^TM^ PMMA, 950,000 molecular weight, Series A7), and Microposit^TM^ S1805^TM^ were purchased from MicroChem corp.

SWCNT micro-lines were obtained on SiO_2_/Si substrate through template guided fluidic assembly. The SiO_2_/Si substrate was first subjected to plasma treatment to improve its affinity to SWCNT-DI water solution. Then photoresist (Microposit^TM^ S1805^TM^) was spin-coated on the SiO_2_/Si and then patterned into arrays of micro-trenches. Finally, the substrate was vertically dip-coated into 0.23 wt.% SWCNTs-DI water solution and gradually lifted at a controlled pulling velocity, V* *= 0.1 mm min^−1^.The SWCNTs were selectively deposited into micro-trenches with exposed SiO_2_ surface. The photoresist was stripped by fresh acetone and isopropanol.

Micro-patterned PDMS substrates were obtained by molding from a bas-relief master mold. The master molds were made of SU-8 (2007) photoresist on SiO_2_/Si substrate by optical lithography. The SU-8 (2007) photoresist was spin coated (Laurel Spinner) on SiO_2_/Si substrate at 500 rpm for 20 s with acceleration of 100 rpm/s and then ramped up to 6000 rpm for 60 s with acceleration of 330 rpm/s. The pre-bake was processed at 65 °C for 1 min and then 95 °C for 2 min. Exposure was performed using Quintel 4000 Mask Aligner with UV filter for 60 s. The post-bake was processed at 65 °C for 1 min and then 95 °C for 2 min. The mold was developed in SU-8 developer for 1 min followed by rinsing in fresh isopropyl alcohol. The thickness of SU-8 structure for the current receipt is 6 μm. PDMS was fabricated by mixing the monomer with curing agent at the ratio of 10: 1 and then casting uncured PDMS into a stainless steel mode. The baking temperature was 115 °C and the baking time was 8 min for the PDMS substrate with a thickness of 5 mm. Unlike the free-standing PDMS substrate, SU-8 substrates were prepared on a supporting substrate (Si, SiO_2_, or glass). To prevent any possible damage from the etching solution (HF), a continuous protective layer SU-8 2007 was spin coated on the surface of supporting substrate first. The same procedures were applied as previously mentioned except that no mask was applied during the exposure and there was no developing process. The micro-patterns of SU-8 were fabricated as the second layer following the exact procedures described in the master mold fabrication. For SEM imaging, all the suspended SWCNT-polymer architectures were coated with a very thin layer (several-nanometer thick) of Au/Pd to eliminate the charging of polymer except for the cases where metal layer is deposited before the transferring process or mentioned otherwise. The tipless cantilever (TL-NCL) for mechanical and electromechanical measurement was obtained from Nanosensors, Inc. It was made of highly doped N-type Si, R = 0.01–0.02 Ω.cm, with 140 nm thick SiO_2_ oxidization layer.

Scanning electron microscopy was performed using Supra 25 from Carl Zeiss AG. Raman microscopy (532 nm laser was applied with 1800 grating) was performed using Jobin Yvon LabRam 800 Raman Spectroscopy system from Horiba Ltd. Transmission electron microscopy (TEM) and Energy-dispersive X-ray spectroscopy (EDX) were performed using JEOL 2100 Field Emission Gun Transmission Electron Microscope (under 200 kV) from JOEL Ltd.

## Additional Information

**How to cite this article**: Li, B. *et al.* Printing Highly Controlled Suspended Carbon Nanotube Network on Micro-patterned Superhydrophobic Flexible Surface. *Sci. Rep.* **5**, 15908; doi: 10.1038/srep15908 (2015).

## Supplementary Material

Supplementary Information
