# Supplementary material for: Printing Highly Controlled Suspended Carbon Nanotube Network on Micro-patterned Superhydrophobic Flexible Surface
Source: Sci Rep. 2015 Oct 29;5:15908. doi: 10.1038/srep15908 (PMC4625127; doi:10.1038/srep15908)
Supplement: Supplementary Information [file srep15908-s1.pdf]

Supporting Information

Printing Highly Controlled Suspended Carbon Nanotube Network on Micro-patterned Superhydrophobic Flexible Surface

Bo Li[1,2], Xin Wang[1], Hyun Young Jung[1,3,4], Young Lae Kim[1], Jeremy T Robinson[5], Maxim Zalalutdinov[5], Sanghyun Hong[1], Ji Hao[1], Pulickel M. Ajayan[2], Kai-Tak Wan[1], Yung Joon Jung[1,3]*

[1]Department of Mechanical and Industrial Engineering, Northeastern University, Boston, MA 02115, USA,
[2] Department of Materials Science and NanoEngineering, Rice University, Houston, TX 77005, USA
[3] George J. Kostas Research Institute for Homeland Security, Northeastern University, Boston, MA 02115, USA
[4]Department of Energy Engineering, Gyeongnam National University of Science and Technology, Jinju, Gyeongnam, 660-758, South Korea
[5]Naval Research Laboratory, Washington, D.C. 20375, USA,

* E-mail: jungy@coe.neu.edu

Table of contents

Characterizations of SWCNTs: Raman spectrum, TEM and EDX

Bending measurement

Probing the solid-liquid-vapor interface on micro-patterned substrates

Mechanical and electromechanical measurements of suspended SWCNT micro-lines

Characterizations of SWCNTs

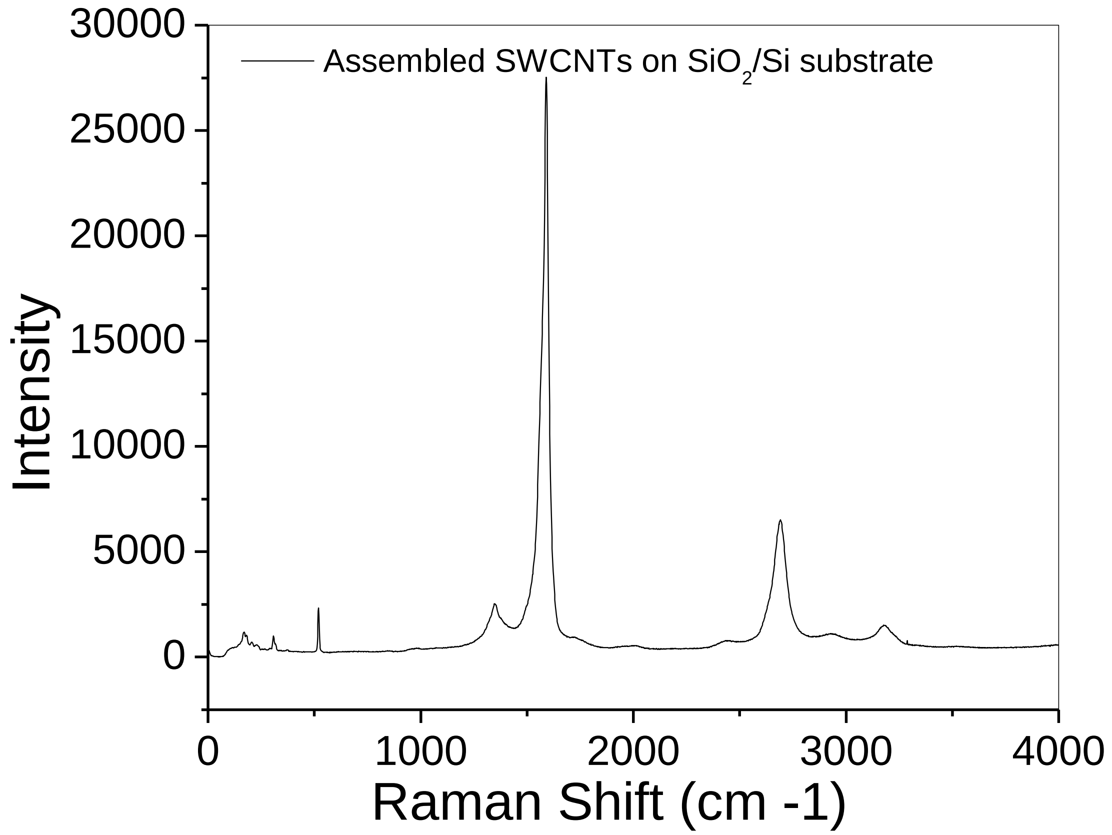

Figure S1 │Raman spectrum of SWCNT micro-lines assembled on SiO_2/Si substrate.

Raman microscopy (532 nm laser was applied with 1800 grating) was performed using Jobin Yvon LabRam 800 Raman Spectroscopy system from Horiba Ltd. The D band and G band are located at 1347 cm^{-1} and 1590.07 cm^{-1}, respectively. The small D/G intensity ratio (~0.1) and G'/G ratio (~0.1) suggest very good quality of carbon nanotubes.

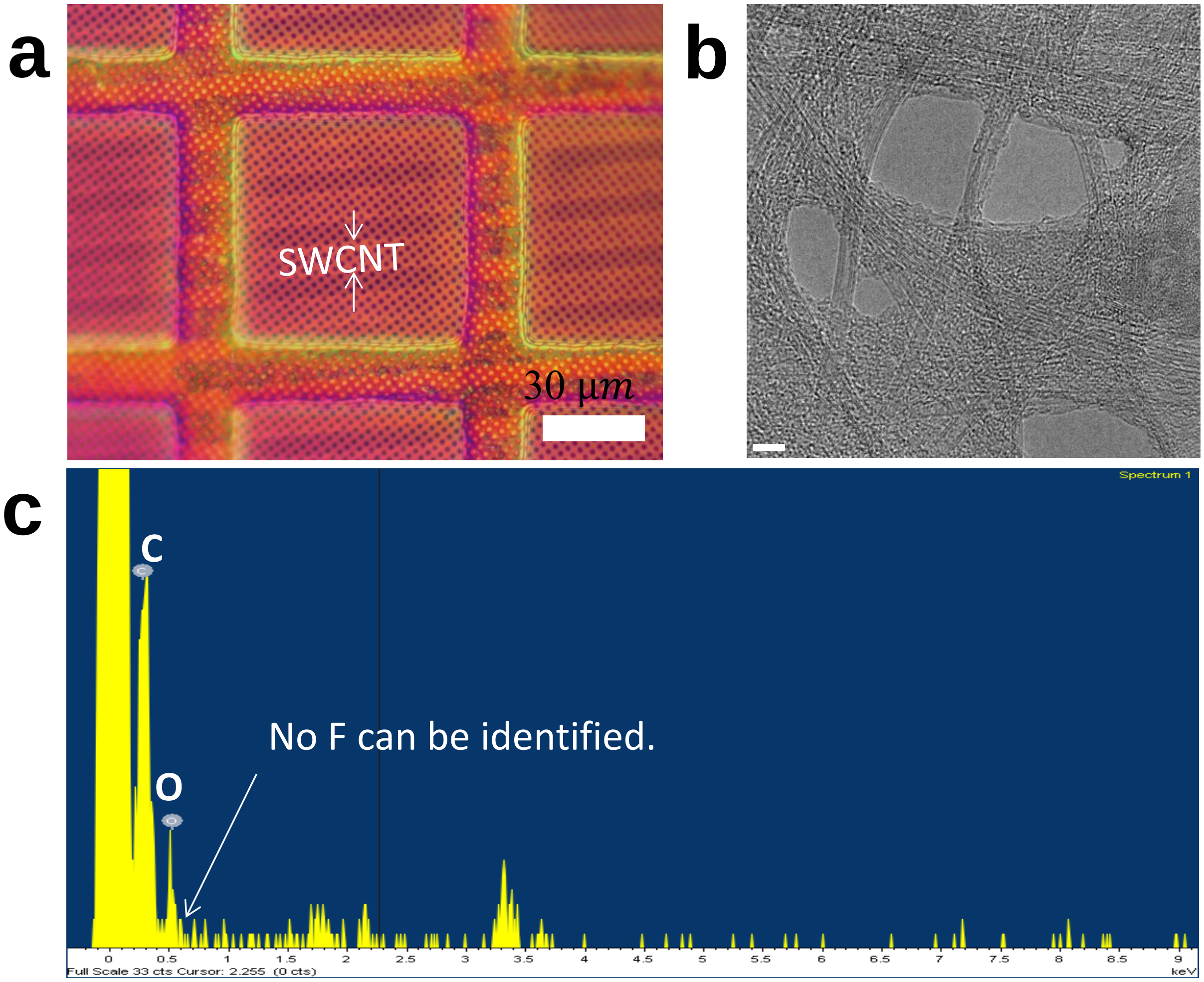

Figure S2│ TEM and EDX characterizations of SWCNT micro-lines. (a)Optical microscopy image of SWCNT micro-lines (9 μm in width) transferred to perforated TEM grid using the identical wet-contact printing process. (b) TEM image of SWCNT network. (c) EDX of suspended SWCNT film and no F can be detected.

In order to investigate the potential influence of HF etching on the properties of SWCNTs, we transferred SWCNT micro-lines to Au TEM grid (Fig. S2a) with perforated holes following the same wet-contact printing recipe. We have performed transmission electron microscopy (TEM) and Energy-dispersive X-ray spectroscopy (EDX) on the same area of the suspended SWCNT film as shown in Fig. S2b and S2c. The result shows no detectable fluorine. We believe

that because the etching procedure is very short (1-2 min), HF cannot create significant doping effect on SWCNTs.

Bending measurement

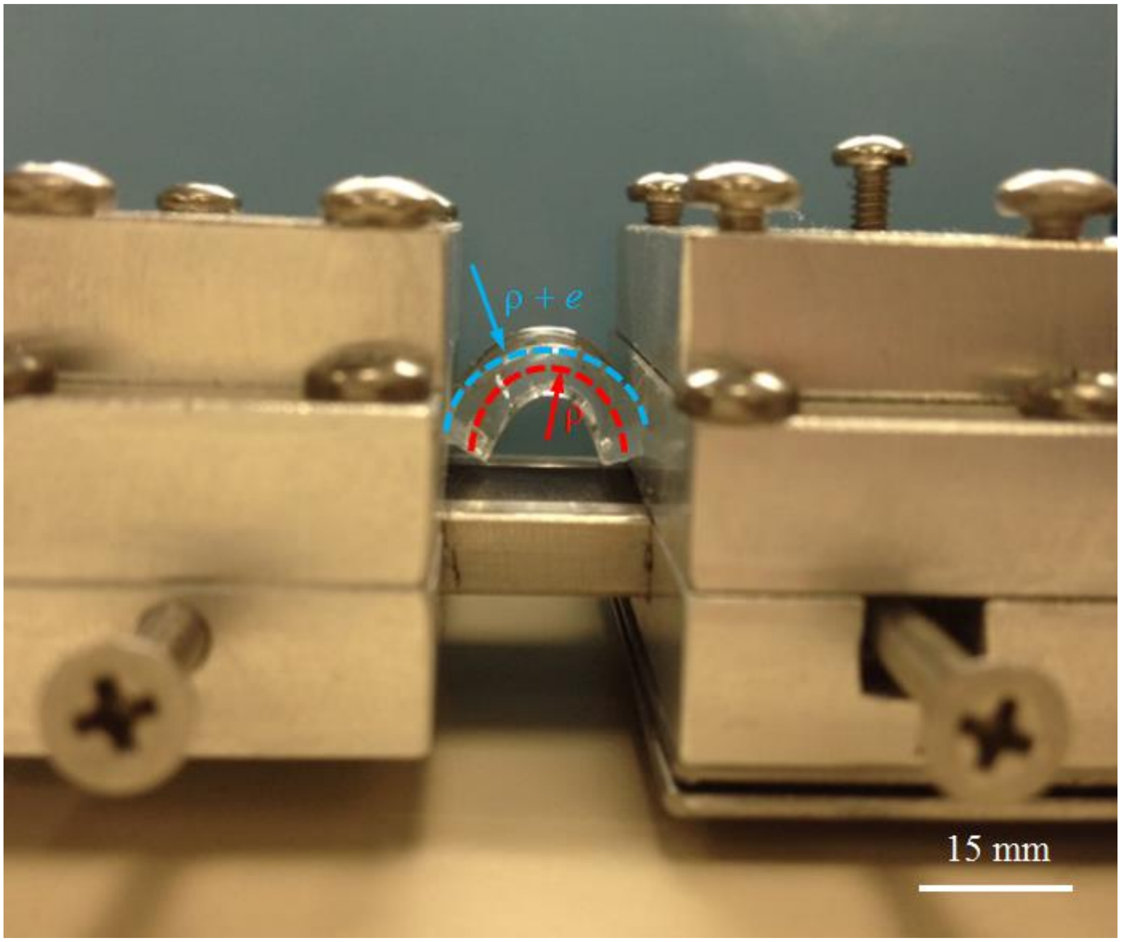

Figure S3 | Bending measurement performed on a customized holder. ρ is the radius of neutral axis after bending. ρ + ε is the radius of the top surface on bulk PDMS substrate.

We have performed the bending test using a customized holder as shown in Fig. S3. The PDMS substrate has a dimension of 13 mm x 2.5 mm (size view). The bending was performed along the PDMS micro-lines to apply a uniform deformation. To simplify the force apply situation, the PDMS bulk with SWCNT network mechanical deformation can be considered as Euler–Bernoulli beam. ρ is the radius of neutral axis after bending. ρ + ε is the radius of the top

surface on bulk PDMS substrate. Based on calculation, the SWCNT network strain is ε = 0.26. We have bent the sample till this strain for 5 times and then released the sample and took the optical image. We did not see large cracks and broken parts of the SWCNT film.

Probing the solid-liquid-vapor interface on micro-patterned substrates

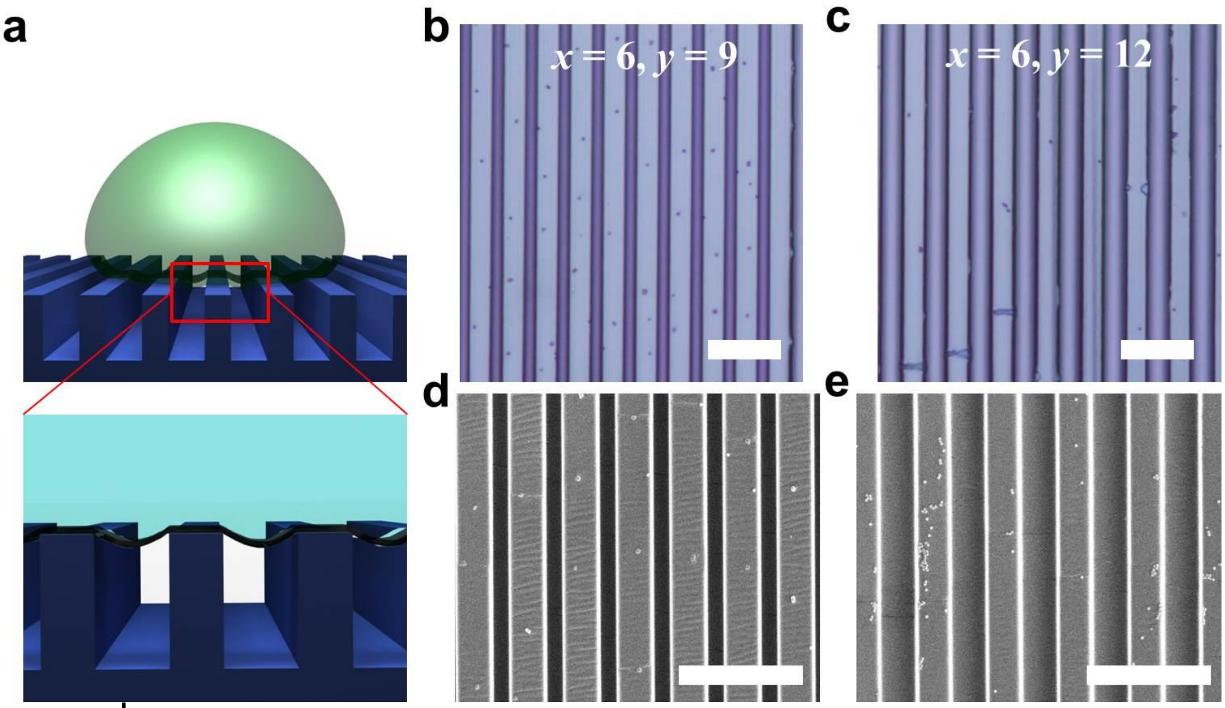

Figure S4 | The formation of Cassie-Baxter interface at the interface between SWCNTs, substrate and solution. (a) The schematic of Cassie-Baxter interface. (b) and (d) The optical and SEM images of nanoparticles on micro-patterned PDMS substrate with $x = z = 6$ μm, and $y = 9$ μm. (c) and (e) The optical and SEM images of nanoparticles on micro-patterned substrate with $x = z = 6$ μm, and $y = 12$ μm. The top surfaces of PDMS micro-strips are shown as brighter bands in both optical and SEM images. The particles are shown as black dots in optical images and white dots in SEM images. The scalar bars are 20 μm for (b) to (e).

The physics governing the formation of suspended SWCNT networks on polymeric patterned substrates is centered on the superhydrophobicity of the receiving substrate. A superhydrophobic surface with nano/micro-patterns is characterized by its wetting behavior,

where only the top surfaces of the supporting patterns are wetted by the solution leaving air pockets between solution and substrate forming a solid-liquid-vapor (SLV) interface (Fig. S4a)[1,2] . This unique SLV interface (between the solution and micro-patterned superhydrophobic substrate) is composed of both solid-liquid interfaces and liquid-air interface, which is known as the heterogeneous interface or the Cassie-Baxter interface[3]. Under this condition water-droplets can easily move over patterned surface without being pinned, analogous to a water droplet on a Lotus leaf. The SWCNT networks can be placed at the SLV interface during this wet contact printing process. Since the liquid solution does not intercalate into the "well" regions, suspended SWCNTs are readily formed by removing remained solutions across the surface. To further investigate the formation of Cassie-Baxter interface, fluorescent nanoparticles (Green fluorescent, ~450 nm in diameter, Duke Scientific Corp.) were mixed into the HF acid solution and deposited on two differently patterned PDMS substrates shown in Fig. S4 (x = 6 and y = 9 and 12 μm). As shown in Fig. S4b-e, fluorescent nanoparticles were observed only on the top surfaces of PDMS micro-line patterns after the transfer process indicating the effective suspension of the solution on the polymer patterns.

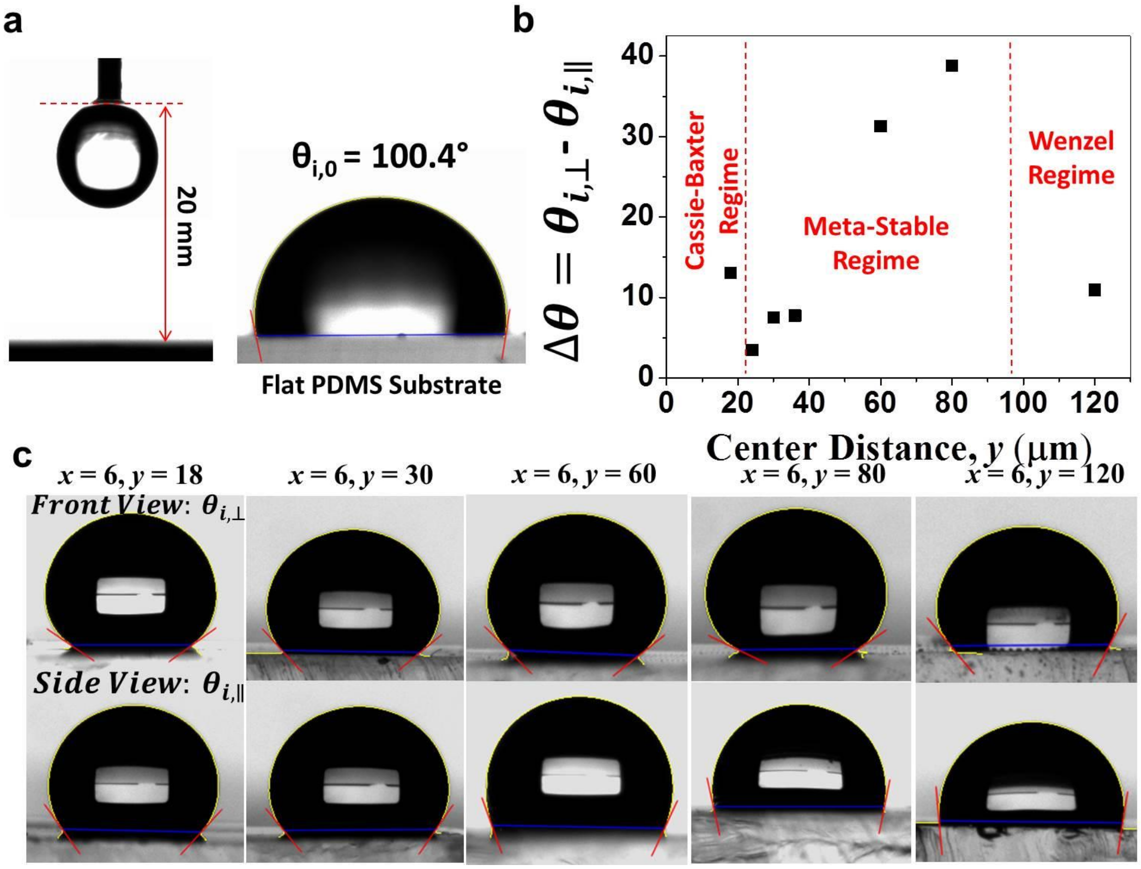

Figure S5 | Contact angle measurement of flat PDMS substrate and micro-patterned PDMS substrate (x = z = 6 μm, and y = 18 to 120 μm). (a) Schematic of contact angle measurement with elevated droplet (height = 20 mm) and representative contact angle measurements on flat PDMS substrates ($\boldsymbol{\theta_{i,0}} = \mathbf{100.4}$°). (b) The dependence of wetting anisotropy ($\Delta\boldsymbol{\theta} = \boldsymbol{\theta_{i,\perp}}$- $\boldsymbol{\theta_{i,\parallel}}$) on center distance, y. (c) Representative contact angle measurements on micro-patterned PDMS substrates (x = z = 6 μm, and y = 18 to 120 μm).

The contact angle measurements with elevated droplets were schematically shown in Fig. S5a, left, where the droplet was elevated 20 mm above the substrate (from the head of needle to the substrate). The purpose of elevating the droplet is to mimic the influence of disturbance on SLV interface during the transfer process. A transition from Cassie-Baxter interface to Wenzel interface can be initiated by inputting external energy in case Cassie-Baxter interface is formed first, *e.g.*, pressing the droplet[4] and elevatingthe droplet to certain height and then releasing[5]. The

transfer process is a dynamic process where the disturbance from sample handling can strongly influence the SLV interface. By elevating droplet to a constant height, the input energy can be fixed, and its influence on the SLV interface can be compared along with the changing geometry of the substrate, though it is not an ideal simulation of real transfer process. The average true contact angle, $\theta_{i,0}$, is 100.2 ± 2.7° for flat PDMS reference surface molded towards a flat SiO_2/Si chip. The wetting anisotropy ($\Delta\theta = \theta_{i,\perp}$ - $\theta_{i,\parallel}$) is summarized in Fig. S5b and its changing tendency can be observed immediately. The typical optical images of contact angle measurement were summarized in Fig. S5c.

When $\mathrm{y} \leq 20$, the strong confinement on solution in the perpendicular direction also hinder the propagation along the parallel direction ($\theta_{i,\perp}$ and $\theta_{i,\parallel}$ are close to each other). The droplets are held tightly over the supporting features forming stable heterogeneous interface. Therefore, nearly 100 % yielding for suspended SWCNT network can be expected as shown in Fig. 2c. This regime can be defined as Cassie-Baxter regime, the best working window for wet-contact printing. However, from y = 20 to 80, $\theta_{i,\parallel}$ diverges from $\theta_{i,\perp}$. This regime can be defined as meta-stable regime evidence by missing and collapsed SWCNT. The meta-stable regime is a state where suspended SWCNT network is possible though 100% yielding is not expected. The sample with y = 120 is located in an unstable regime for Cassie-Baxter interface (or stable regime for Wenzel interface), where $\theta_{i,\perp}$ approaches $\theta_{i,\parallel}$ again but ending at Wenzel interface ($\theta_{i,Wenzel}$) and no suspended SWCNT can be obtained.

Mechanical and electromechanical measurements of suspended SWCNT micro-lines

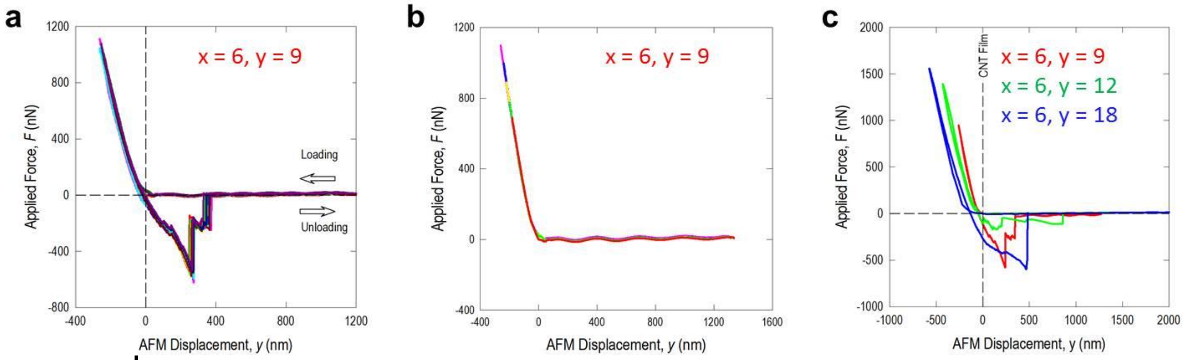

Figure S6│Mechanical measurements on suspended SWCNT micro-lines transferred to SU-8 substrates patterned with micro-lines (*x* = *z* = 6 and *y* = 9, 12, 18 μm). (a) Ten identical indentation loops on the SWCNT micro-lines transferred to *y* = 9 substrate, (b) five loading curves with different indentation depth on the same sample and (c) indentation on samples SWCNT micro-lines transferred to substrates with different center distance, *y*.

Tipless cantilever (TL-NCL, Nanosensors, Inc.; highly doped N-type Si, R= 0.01-0.02 Ω.cm) is formed by milling off the cantilever using a focused ion beam (FIB). To avoid dissipating charge on the SWCNT film from the cantilever, we oxidized the cantilever in air at temperature (1000 °C, *t* = 5 hrs) to form a 140-nm-thick SiO_2 layer. For mechanical measurement, arrays of suspended SWCNT micro-lines (about 6 μm in width) have been transferred to SU-8 substrate patterned with micro-lines (*x* = *z* = 6 and *y* = 9, 12, 18 μm). The width of supporting strip, *x*, is 6 μm and the center distance, *y*, ranges from 9 to 18 μm. The height of the SU-8 micro-line, *z*, is also 6 μm leaving enough space for the indentation process. Fig. S6a shows ten identical indentation loops on SWCNT micro-lines transferred to *y* = 9 substrate and great repeatability can be found during different indentation and contraction loops. Fig. S6b shows overlapping of indentation loops with five different indentation depths. Three representative loops of three substrate designs (*y* = 9, *y* = 12 and *y* = 18) are shown in Fig. S6c. The established V-peel model[6] is used to fit the SWCNT thin film mechanical response with elastic modulus, *E*, and average thickness, *h*.

Generally speaking, an external line load, F, is applied at the centerline of a strip with width, b, length, $2l$, thickness, h, elastic modulus, E and Poission's ratio, ν, which is adhered to a substrate. The film is elastically deformed into a V-shape under a mixed bending and stretching. The film profile is denoted by $w(x)$ with a central deflection, w_0. For simplicity, a set of useful dimensionless parameters are defined as follows:

$$\xi = \frac{x}{l}, \qquad \omega = \frac{w}{h}, \qquad \omega_0 = \frac{w_0}{h}, \qquad \beta = l\left(\frac{N}{D}\right)^{1/2}, \qquad \varphi = \frac{Fl^3}{2Dbh} \qquad \textbf{(S1)}$$

The parameter β is defined to be the ratio of membrane stress to film rigidity such that β≈0 for pure bending and β→∞ for pure stretching. The final analytical solution is

$$\omega_0 = \frac{\varphi}{\beta^3}\left[-\sinh\beta + \frac{(\cosh\beta - 1)^2}{\sinh\beta} + \beta\right] \qquad \textbf{(S2)}$$

$$\varphi = \sqrt{\frac{\beta^7(1+\cosh\beta)}{6(2\beta + \beta\cosh\beta - 3\sinh\beta)}} \qquad \textbf{(S3)}$$

Raw AFM force-displacement (F vs. y) is converted to the relation between applied force and thin film deflection at the centerline (F vs. w_0) by correcting for cantilever deflection from the piezo displacement. Two apparent dominant regions are shown in the log-log plot of F vs. w_0. The stretching dominance with relation of $F \propto w_0^3$ yields a linear dependence of log [F] upon log [w_0] with slope of 3 and bending deformation mode gives rise to a linear F (w_0). The elastic modulus, E, and thin film average thickness, h, are deduced from the intercepts of two linear regions with F-axis.

Figure 4b is the applied force vs. thin film central deflection in the bending region. The transition happens when ω_0 reaches $\sqrt{2}$.

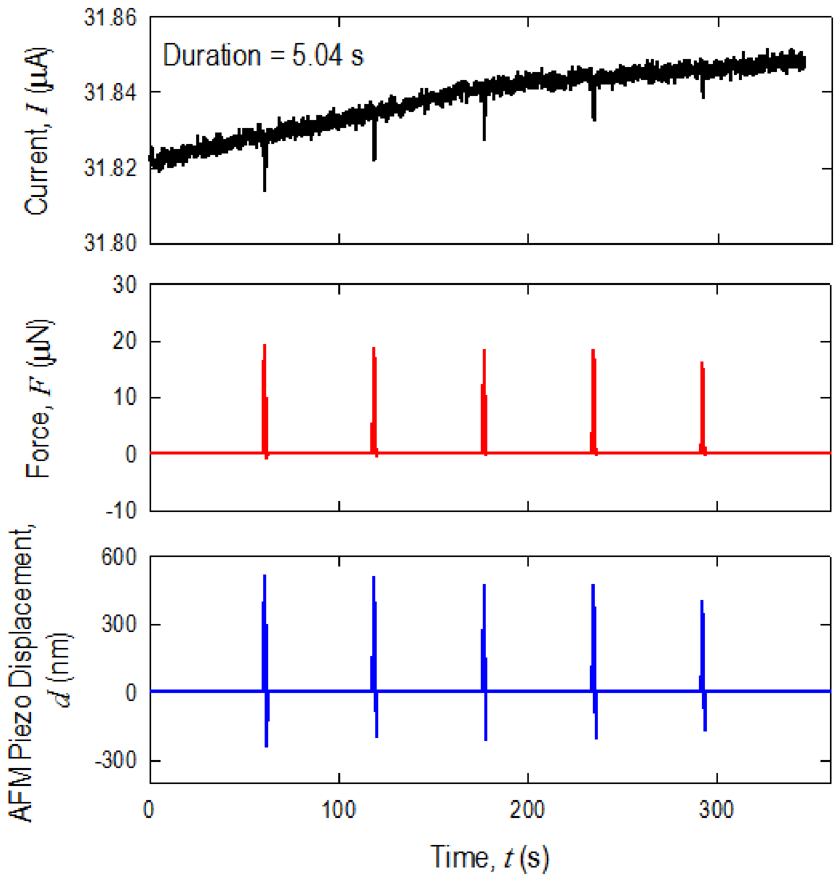

Figure S7 | A representative five-time electromechanical measurement on one suspended SWCNT micro-line. The dependence of current (a), force (b) and deformation (c) on time.

The sample for electromechanical measurement was made by transferring SWCNT micro-lines (10.7 μm wide) to SU-8 patterned transparent glass substrate. Pd electrodes (50 nm thick) were deposited on the substrate. To achieve better thickness uniformity of SWCNT film, the sample for electromechanical measurement has been fabricated by O_2 plasma etching of a whole film assembled on SiO_2 substrate. The average thickness of the SWCNT film is measured to be 45.7 nm. In order to keep only one thin film connected between two electrodes, a micro-capillary

needle controlled by high resolution micro-manipulator was used to break the other thin films along the trench edge. The sample was then integrated into a customized electromechanical measurement set-up composed of an AFM (to deform the film) and Keithley 2400 (to collect the change of current). The loading-unloading cycle ran with a certain duration time (performed by AFM) and the current passing though the SWCNT sample of the SWCNT thin film was simultaneously recorded as a function of time (by Keithley 2400). Figure S7 shows a representative set of 5-time repeated electromechanical measurements on the same suspended SWCNT thin film. The first panel (Fig. S7a) records the current as a function of time. An immediate drop of current can be observed once the cantilever starts to deform the suspended SWCNT micro-line. The absolution value of the lowest point of current subtracted by the base line is termed as ΔI. The second (Fig. S7b) and the third panel (Fig. S7c) record the force and deformation sensed by AFM. Based on the geometry and the AFM piezo displacement (*d*), it is straightforward to calculate the vertical indentation depth of suspended thin film at the central line, based on which the thin film length change Δl is measured. Current was monitored in 6 minutes and indentations were performed at each end of the first 5 minutes. Multiple samples were fabricated and characterized using the same electromechanical method. Twenty different measurements from different samples were used to define the fitting curve in Fig. 4c. The external voltage, *U*, was 0.1 V SWCNT samples. 5.04 s was used as duration times for AFM indentation to investigate the electromechanical hysteresis response.

The electrical conductivity was calculated based on the curve fitting to ΔI vs. Δl with R^2 of 0.99. The detailed calculations are shown as follows:

$$G = \frac{I}{U} = \sigma \frac{A}{l}, \tag{S4}$$

where *G, I, U, σ, A, l* are conductance, current, voltage, conductivity, cross section area and length of the film between contact pads. Change of current, which can be directly obtained in the measurement, can be expressed as

$$\Delta I = |I_1 - I_0| = |U(G_1 - G_0)| = \left|U\sigma(\frac{A}{l_1} - \frac{A}{l_0})\right| = U\sigma A \left|\frac{l_1 - l_0}{l_1 l_0}\right|, \tag{S5}$$

where subscripts 1 and 0 indicate the status during indentation (1) and the initial status before indentation (0). Since the change of length is small, the expression for ΔI can be simplified as:

$$\Delta I = \frac{\Delta l}{l_0^2} U\sigma A, \tag{S6}$$

The slope from the fitted curve in Fig. 4c can be expressed as:

$$Slope = \frac{1}{l_0^2} U\sigma A. \tag{S7}$$

As shown in Fig. 4c, the slopes for 10.7-µm-wide suspended SWCNT film is 0.66. l_0 is 489 µm and U is 0.1 V. Therefore, the conductivity can be calculated from Equation S7 as 3.2×10^6 S m^{-1}. The conductivity of two terminal measurement calculated from Equation S4 is 3.2×10^5 S m^{-1} given the value I = 31.8 µA. Considering the fact that electromechanical measurement (calculation from Equation S7) could exclude the influence of contact resistance, 3.2×10^6 S m^{-1} would be more accurate. Most importantly, the linear dependence of ΔI on deformation (Δl) indicates a very stable and sensitive device for immediate applications in displacement sensing and force sensing.

References

1 Neinhuis, C. & Barthlott, W. Characterization and distribution of water-repellent, self-cleaning plant surfaces. *Ann. Bot.* **79**, 667-677 (1997).
2 Quéré, D. Wetting and roughness. *Annu. Rev. Mater. Res.***38**, 71-99 (2008).
3 Cassie, A. & Baxter, S. Wettability of porous surfaces. *T. Faraday Soc.* **40**, 546-551 (1944).
4 Callies, M. & Quéré, D. On water repellency. *Soft Matter* **1**, 55-61 (2005).
5 He, B., Patankar, N. A. & Lee, J. Multiple equilibrium droplet shapes and design criterion for rough hydrophobic surfaces. *Langmuir* **19**, 4999-5003 (2003).
6 Wan, K. T. Fracture mechanics of a V-peel adhesion test - Transition from a bending plate to a stretching membrane. *J. Adhesion* **70**, 197-207 (1999).
